# Sleep quality in middle-aged and elderly Chinese: distribution, associated factors and associations with cardio-metabolic risk factors

**DOI:** 10.1186/1471-2458-9-130

**Published:** 2009-05-09

**Authors:** Nazanin Haseli-Mashhadi, Tony Dadd, An Pan, Zhijie Yu, Xu Lin, Oscar H Franco

**Affiliations:** 1 Unilever R&D, Colworth Science Park, Sharnbrook, Bedfordshire, MK441LQ, UK; 2 Key Laboratory of Nutrition and Metabolism, Institute for Nutritional Sciences, Shanghai Institutes for Biological Sciences, Chinese Academy of Sciences and Graduate School of the Chinese Academy of Sciences, Shanghai, PR China; 3 Health Sciences Research Institute, Warwick Medical School, The University of Warwick, Coventry, CV47AL, UK

## Abstract

**Background:**

Poor sleep quality has been associated with increased risk of heart disease, diabetes and mortality. However, limited information exists on the distribution and determinants of sleep quality and its associations with cardio-metabolic risk factors in Chinese populations. We aimed to evaluate this in the current study.

**Methods:**

A cross-sectional survey conducted in 2005 of 1,458 men and 1,831 women aged 50–70 years from urban and rural areas of Beijing and Shanghai. Using a questionnaire, sleep quality was measured in levels of well, common and poor. Comprehensive measures of socio-demographical and health factors and biomarkers of cardio-metabolic disease were recorded. These were evaluated in association with sleep quality using logistic regression models.

**Results:**

Half of the population reported good sleep quality. After adjusting for potential confounders, women and Beijing residents had almost half the probability to report good sleep quality. Good physical and mental health (good levels of self-rated health (OR 2.48; 95%CI 2.08 to 2.96) and no depression (OR 4.05; 95%CI 3.12 to 5.26)) related to an increased chance of reporting good sleep quality, whereas short sleep duration (<7 hrs OR 0.10; 95%CI 0.07 to 0.14)) decreased it substantially. There were significant associations between levels of sleep quality and concentrations of plasma insulin, total and LDL cholesterol, and index of insulin resistance.

**Conclusion:**

Levels of good sleep quality in middle-age and elderly Chinese were low. Gender, geographical location, self-rated health, depression and sleep quantity were major factors associated with sleep quality. Prospective studies are required to distil the factors that determine sleep quality and the effects that sleep patterns exert on cardio-metabolic health.

## Background

Sleep disturbances, including chronic insomnia (difficulty in maintaining sleep, having intermittent awakening and early awakening) and sleep apnea are increasingly common in modern societies with reported prevalences varying between 4 and 22 percent in different populations.[1] Sleep disturbances are common in later life, with loss of nocturnal sleep continuity and depth. [2-4] This could be due to the high prevalence of diseases accompanied by pain and stress, the side effects of taking medications and the inherent changes in sleep architecture that tend to occur with age. Aside from age and gender, physical and mental health problems are reported to be significantly associated with sleep disturbances. [5-8] Across different populations, several studies have found a significant deleterious effect of sleep disturbances on self-rated health, incidence of cardio-metabolic diseases (coronary heart disease, metabolic syndrome and type 2 diabetes), quality of life and mortality. [9-25] Various mechanisms have been explored and suggested as the potential link between poor sleep patterns and negative health outcomes (e.g. impaired glucose regulation and subsequent increased risk of type 2 diabetes[24]). Nevertheless the existing information is limited and the pathophysiological pathways between poor sleep patterns and cardio-metabolic disease remain unclear. Furthermore, although ample data exist for different populations, there is limited information on the levels of sleep disturbances among Chinese populations, the possible factors associated and the potential association of sleep quantity and quality with biomarkers of early cardio-metabolic disorders.

Therefore, in order to evaluate the levels of sleep patterns in Chinese and contribute to complement its related gap of epidemiological evidence and knowledge, in the current study we aimed to evaluate: (i) the distribution of sleep quality; (ii) factors that determine sleep quality; and (iii) whether self-reported sleep quality is associated with biomarkers of cardio-metabolic disease in middle-aged and elderly Chinese.

## Methods

### Study population

We used data from "Nutrition and Health of Aging Population in China" study. [26-28] This is a cross-sectional survey conducted between March and June 2005, among 3289 subjects (1,458 men and 1,831 women) aged 50 to 70 years from urban and rural areas of Beijing and Shanghai (metropolitan cities of north and south of China). The sampling was conducted with an emphasis on recruiting at least 40% of men, a similar number of rural/urban residents of Beijing and Shanghai and a representation of people from all levels of education and income. Trained physicians or public health workers completed the sampling through a face-to-face interview using a standardized questionnaire to collect socio-demographical, lifestyle and health related information. Further details about the questionnaire can be found elsewhere.[29]

The study protocol was approved by the Institutional Review Board of the Institute for Nutritional Sciences. All participants provided written informed consents.

### Assessment of sleep and related factors

Subjective sleep quality during the last month was recorded in 3 levels (well, common or poor). Sleep quantity was measured as self-reported average total hours of daily sleep during the last month, including both night and nap hours sleep and was categorized as under, normal and over (<7, 7 to 9 and >9 hours per day respectively).

Socio-demographic variables included age (50–59 and 60–70 yrs), gender, geographic region (Beijing or Shanghai), residence of living (urban or rural), educational attainment (low, moderate and high, based on the number of years of education as 0 to 6, 7 to 9, and ≥10 years respectively), total household income (low, moderate and high on the basis of less than 10,000, 10,000 to 29,999 and more than 30,000 Yuan annually), marital status (married or without spouse), employment status (grouped as currently employed, unemployed/on welfare system and retired) and social activity ('yes' or 'no' on the basis of participating in at least one of a list of social activities such as painting, playing chess, singing and dancing).

Lifestyle variables included smoking habit, defined as never, current and former (cessation of smoking >6 months); alcohol drinking (grouped into "yes "or "no") and physical activity level (low, moderate, or high) according to the IPAQ (International Physical Activity Questionnaire).

Self rated health (SRH) was initially recorded in 5 levels (very good, good, fair, poor and very poor) and then combined into two categories: good (very good and good) and poor (fair, poor and very poor).[30,31] Pulmonary disease was defined as existence of at least one of the following: asthma, chronic bronchitis, emphysema or chronic cor-pulmonale diseases. Presence of diabetes was defined as either measured fasting glucose greater than 7.0 mmol/L or being diagnosed with type 2 diabetes or taking anti-diabetes drugs or insulin. Presence of cardiovascular disease (CVD) was defined as one or more definite manifestations of coronary heart disease or stroke.

The Center for Epidemiologic Studies Depression Scale (CES-D)[32], which has been previously validated in Chinese populations[33,34], was used to measure the presence of clinically relevant depressive symptoms. Using the accepted cut-off point of 16 for the sum of scores, a CES-D score of 0–15 was defined as no or minimal depression and a CES-D score ≥16 was defined as minor depression.[32] Following a physical examination, BMI levels were measured and categorized as under weight (<18.5 kg/m^2^), normal weight (18.5 to <24.0 kg/m^2^) and overweight/obese (≥24.0 kg/m^2^).[35] Three measurements of blood pressure were taken using an electronic blood pressure monitor and the mean of the last two measurements was used for analysis.

### Laboratory methods

Overnight fasting blood samples were collected in tubes containing liquid ethylenediaminetetraacetic acid (EDTA), centrifuged at 4°C, and stored at -80°C until analysis. Total cholesterol, high-density lipoprotein (HDL) cholesterol, low-density lipoprotein (LDL) cholesterol, triglycerides (TG), and glucose were measured enzymatically (Hitachi 7080, Japan). Plasma C-reactive protein (CRP) was measured by a particle-enhanced immunoturbidimetric assay (Ultrasensitive CRP kit, Orion Diagnostica, Espoo, Finland). Plasma adiponectin concentrations were measured by Luminex xMAP™ Technology (Linco Research Inc, St Charles, Mo). All procedures followed the manufacture's instruction with quality controls in the expected ranges for each assay. Human Retinol Binding Protein 4 (RBP4) was measured by a sandwich ELISA developed in-house.[36] Interleuikin 6 (IL-6) was measured with high-sensitivity enzyme-linked immuno-sorbent assay (ELISA) (Quantikine HS IL-6 Immunoassay, R&D Systems, Inc., Minneapolis, Minnesota). Plasma adiponectin, resistin and active plasminogen activator inhibitor-1 (PAI-1) concentrations were measured by Luminex xMAPTM Technology (Linco Research Inc, St Charles, Mo).[27] Fasting glucose was measured enzymatically on an automatic analyzer (Hitachi 7080, Japan). Fasting insulin was determined by radioimmunoassay (Linco Research, MO). Index of insulin resistance was calculated using updated homeostasis model assessment methods (HOMA2-IR, using the HOMA2 calculator, ).[37]

### Statistical analyses

We evaluated the distribution of sleep quality in the total population and how it varied by gender, geographic region and area of residence. Using a chi-square test, analysis of variance or Wilcoxon rank-sum test and univariate analysis, we evaluated the associations between sleep quality and potential associated factors (selected based on previous literature or scientific knowledge). Factors that significantly modified sleep quality or those that were consistently reported as significant determinants in previous publications were selected to be included in multivariate analysis applying an ordinal regression model. Multivariate analysis was repeated stratifying by gender, region and residence one at a time and by stratifying for two of these factors simultaneously.

The associations between sleep quality and biomarkers of cardio-metabolic disease were tested on the total population and on a sub-sample of participants whom were free from CVD and diabetes with parametric t-tests (after logarithmic transformation of the data to satisfy normality assumptions) or non-parametric Wilcoxon rank-sum tests.

Data management and statistical analyses were performed with the SAS statistical package version 9.1 (SAS Institute, Cary, NC). Statistical tests were two-sided and at a 5% level of significance.

## Results

### Characteristics of the participants

The mean age of the study sample was 58.6 years (Table 1). More than half of the participants were women (55.7%), 72% were non-smokers (94% women and 44% men), 72% were non alcohol drinkers (90% women and 48% men), 92% were involved in moderate or high levels of physical activity and 53% were overweight or obese. Three percent of the participants reported taking sleeping pills (4% women and 1% men), 42% reported taking a nap (56% in Beijing, 29% in Shanghai), 68% reported having 7–9 hours of daily sleep and 53% reported good sleep quality (Table 1). Thirty two percent of participants reported good/excellent self-rated health, 9% of participants suffered from depressive symptoms (15% in Beijing and 4% in Shanghai), 14% had diabetes (19% in Beijing and 9% in Shanghai), 10% had CVD (15% in Beijing and 6% in Shanghai), 42% had metabolic syndrome (50% in Beijing and 35% in Shanghai), 14% had hyperlipidemia, 7% had pulmonary disease, 13% arthritis and 11% prostatisim (Table 1).

**Table 1:** Characteristics of the study participants by region

**Variable, N (%)**	**Total**	**Beijing**	**Shanghai**	**P-value**
Female gender	1819 (55.71)	894 (54.91)	925 (56.51)	0.3599
Rural residence	1638 (50.17)	808 (49.63)	830 (50.70)	0.5405
Age, mean(std)	58.59 (6.01)	58.30 (5.95)	58.87 (6.06)	0.0072
Age category, years				0.0134
50≤ age <60	1845 (56.51)	955 (58.66)	890 (54.37)	
60≤ age ≤70	1420 (43.49)	673 (41.34)	747 (45.63)	
BMI, mean(std)	24.46 (3.59)	25.26 (3.65)	23.67 (3.35)	<.0001
BMI category				<.0001
Under weight	111 (3.40)	37 (2.27)	74 (4.52)	
Normal	1411 (43.22)	570 (35.01)	841 (51.37)	
Over weight/Obese	1743 (53.38)	1021 (62.71)	722 (44.11)	
Currently married (YES, %)	2862 (87.66)	1407 (86.43)	1455 (88.88)	0.0328
Living alone (YES, %)	185 (5.67)	93 (5.72)	92 (5.62)	0.9056
Smoking				<.0001
Current	910 (27.87)	508 (31.20)	402 (24.56)	
Former	326 (9.98)	206 (12.65)	120 (7.33)	
Never	2029 (62.14)	914 (56.14)	1115 (68.11)	
Current alcohol drinker	930 (28.48)	602 (36.98)	328 (20.04)	<.0001
Physical activity, level				<.0001
Low	242 (7.41)	128 (7.86)	114 (6.96)	
Moderate	1368 (41.90)	581 (35.69)	787 (48.08)	
High	1655 (50.69)	919 (56.45)	736 (44.96)	
Educational level, years in school				<.0001
0–6	1348 (41.29)	562 (34.52)	786 (48.01)	
7–9	1163 (35.62)	698 (42.87)	465 (28.41)	
≥10	754 (23.09)	368 (22.60)	386 (23.58)	
Medical insurance (YES, %)	2280 (70.05)	872 (53.83)	1408 (86.12)	<.0001
Annual income, Yuan				<.0001
<10000	903 (29.07)	396 (25.13)	507 (33.14)	
10000–29999	1409 (45.36)	797 (50.57)	612 (40.00)	
≥30000	794 (25.56)	383 (24.30)	411 (26.86)	
Employment				<.0001
Employed	778 (23.83)	386 (23.71)	392 (23.95)	
Retired	1803 (55.22)	842 (51.72)	961 (58.70)	
Unemployed/on welfare	684 (20.95)	400 (24.57)	284 (17.35)	
Social activities (YES, %)	1661 (50.87)	848 (52.09)	813 (49.66)	0.1659
Self-rated health status (good, %)	1041 (31.90)	483 (29.69)	558 (34.11)	0.0067
Diabetes (YES, %)	446 (14.04)	292 (18.83)	154 (9.48)	<.0001
Metabolic syndrome (YES, %)	1392 (42.63)	815 (50.06)	577 (35.25)	<.0001
Hyperlipidemia (YES, %)	430 (13.78)	257 (17.11)	173 (10.69)	<.0001
Cardiovascular disease (YES, %)	327 (10.24)	228 (14.53)	99 (6.10)	<.0001
Pulmonary disease (YES, %)	238 (7.33)	118 (7.30)	120 (7.37)	0.9399
Arthritis (YES, %)	431 (13.41)	227 (14.29)	204 (12.55)	0.1458
Prostatism (YES, %)	150 (10.81)	77 (11.21)	73 (10.43)	0.6402
Depressive symptoms (YES, %)	307 (9.40)	241 (14.80)	66 (4.03)	<.0001
Sleeping pills intake (YES, %)	97 (2.97)	59 (3.63)	38 (2.32)	0.0281
Having nap (YES, %)	1382 (42.33)	911 (55.96)	471 (28.77)	<.0001
Sleep quality				<.0001
Well	1736 (53.17)	757 (46.50)	979 (59.80)	
Common	1001 (30.66)	558 (34.28)	443 (27.06)	
Poor	528 (16.17)	313 (19.23)	215 (13.13)	
Sleep quantity, total hours of sleep/day				<.0001
<7	799 (24.47)	346 (21.25)	453 (27.67)	
7–9	2208 (67.63)	1119 (68.73)	1089 (66.52)	
>9	258 (7.90)	163 (10.01)	95 (5.80)	

Abbreviations: SD, standard deviation; BMI, Body Mass Index; CI, Confidence interval.

*Data are given as frequency (percentage) unless otherwise indicated.

### Distribution of sleep quality

More than half of the study population (53%) reported good sleep quality (Beijing 46% vs. Shanghai 60%, women 47% vs. 61% men). Almost a third of the population (31%) reported a 'common' level of sleep quality (Beijing 34% vs. 27% Shanghai, women 33% vs. 28% men) and the rest (16%) reported poor sleep quality (19% Beijing vs. 13% Shanghai, women 20% vs. men 11%) (Table 1, data for gender difference not shown).

Eighty-eight percent of the participants who reported good sleep quality reported seven or more hours of daily sleep. In participants that reported poor sleep quality, 84% had fair, poor or very poor SRH. Three per cent of those with good sleep quality reported depressive symptoms, compared to 27% of those who reported poor sleep quality Additionally, higher prevalence of CVD (20% vs. 7%), arthritis (21% vs. 10%) and prostatisim (19% vs. 8%) was observed among those that reported poor sleep quality compared to those with good sleep quality (Table 2).

**Table 2:** Characteristics of the study participants by quality of sleep

**Variable, N (%)**	**Total**	**Well**	**Common**	**Poor**	**P-value**
Female gender	1819(55.71)	849(48.91)	603(60.24)	367(69.51)	<.0001
Beijing region	1628(49.86)	757(43.61)	558(55.74)	313(59.28)	<.0001
Rural residence	1638(50.17)	956(55.07)	420(41.96)	262(49.62)	<.0001
Age, mean(std)	58.59 (6.01)	58.61 (6.05)	58.69(6.00)	58.34(5.90)	0.5617
Age category, years					0.2195
50≤ age <60	1845(56.51)	975(56.16)	554(55.34)	316(59.85)	
60≤ age ≤70	1420(43.49)	761(43.84)	447(44.66)	212(40.15)	
BMI, mean(std)	24.46 (3.59)	24.33 (3.50)	24.66(3.68)	24.53(3.66)	0.056
BMI category					0.1373
Under weight	111 (3.40)	61 (3.51)	27 (2.70)	23 (4.36)	
Normal	1411(43.22)	776 (44.70)	423 (42.26)	212 (40.15)	
Over weight/Obese	1743(53.38)	889 (51.79)	551 (55.04)	293 (55.49)	
Currently married (YES, %)	2862(87.66)	1547(89.11)	858(85.71)	457(86.55)	0.0237
Living alone (YES, %)	185(5.67)	89(5.13)	63(6.29)	33(6.25)	0.3664
Smoking					<.0001
Current	910(27.87)	550(31.68)	241(24.08)	119(22.54)	
Former	326(9.98)	184(10.60)	98(9.79)	44(8.33)	
Never	2029(62.14)	1002(57.72)	662(66.13)	365(69.13)	
Current alcohol drinker	930(28.48)	524(30.18)	293(29.27)	113(21.40)	0.0004
Physical activity, level					0.0025
Low	242(7.41)	120(6.91)	74(7.39)	48(9.09)	
Moderate	1368(41.90)	681(39.23)	455(45.45)	232(43.94)	
High	1655(50.69)	935(53.86)	472(47.15)	248(46.97)	
Educational level, years in school					0.0026
0–6	1348(41.29)	762(43.89)	366(36.56)	220(41.67)	
7–9	1163(35.62)	599(34.50)	371(37.06)	193(36.55)	
≥10	754(23.09)	375(21.60)	264(26.37)	115(21.78)	
Medical insurance (YES, %)	2280(70.05)	1236(71.40)	711(71.24)	333(63.31)	0.0011
Annual income, Yuan					0.0116
<10000	903(29.07)	512(31.05)	239(24.82)	152(30.77)	
10000–29999	1409(45.36)	718(43.54)	469(48.70)	222(44.94)	
≥30000	794(25.56)	419(25.41)	255(26.48)	120(24.29)	
Employment					<.0001
Employed	778(23.83)	473(27.25)	207(20.68)	98(18.56)	
Retired	1803(55.22)	905(52.13)	598(59.74)	300(56.82)	
Unemployed/on welfare	684(20.95)	358(20.62)	196(19.58)	130(24.62)	
Social activities (YES, %)	1661(50.87)	910(52.42)	508(50.75)	243(46.02)	0.0362
Self-rated health status (good, %)	1041(31.90)	754(43.46)	203(20.30)	84(15.91)	<.0001
Diabetes (YES, %)	446(14.04)	211(12.48)	154(15.81)	81(15.85)	0.0255
Metabolic syndrome (YES, %)	1392(42.63)	694(39.98)	451(45.05)	247(46.78)	0.0038
Hyperlipidemia (YES, %)	430(13.78)	175(10.45)	169(17.81)	86(17.30)	<.0001
Cardiovascular disease (YES, %)	327(10.24)	122(7.17)	103(10.59)	102(19.69)	<.0001
Pulmonary disease (YES, %)	238(7.33)	111(6.43)	77(7.73)	50(9.52)	0.0501
Arthritis (YES, %)	431(13.41)	175(10.21)	144(14.69)	112(21.54)	<.0001
Prostatism (YES, %)	150(10.81)	66(7.78)	55(14.36)	29(18.59)	<.0001
Depressive symptoms (YES, %)	307(9.40)	49(2.82)	116(11.59)	142(26.89)	<.0001
Sleeping pills intake (YES, %)	97(2.97)	12(0.69)	21(2.10)	64(12.12)	<.0001
Having nap (YES, %)	1382(42.33)	739(42.57)	428(42.76)	215(40.72)	0.7131
Sleep quantity, total hours of sleep/day					<.0001
<7	799(24.47)	209(12.04)	273(27.27)	317(60.04)	
7–9	2208(67.63)	1348(77.65)	668(66.73)	192(36.36)	
>9	258(7.90)	179(10.31)	60(5.99)	19(3.60)	

Abbreviations: SD, standard deviation; BMI, Body Mass Index; CI, Confidence interval.

*Data are given as frequency (percentage) unless otherwise indicated.

### Sleep quality: associated factors

Based on the results of the univariate analyses -and previous publications-, gender, region, residential status, BMI, alcohol consumption and smoking, physical activity, educational level, marital status, medical insurance coverage, social activity, SRH, diabetes, CVD, arthritis, prostatisim, depressive symptoms and sleep quantity were chosen to be analysed in the multivariate analysis (Table 3).

**Table 3:** Adjusted associated factors of sleep quality for the total population and stratified by regional area of living

	Association with sleep quality (OR with 95% CI)		
**Variable**	**Total**	**Beijing**	**Shanghai**

Gender (female *vs*. male)	0.51 (0.41–0.64)*	0.55 (0.40–0.75)*	0.50 (0.36–0.71)*
Residential location (rural *vs. *urban)	1.27 (1.01–1.60)*	1.08 (0.77–1.52)	1.66 (1.13–2.43)*
Geographic location (Beijing *vs. *Shanghai)	0.61 (0.51–0.73)*		
Body Mass Index (BMI)			
normal vs. over weight	0.92 (0.79–1.07)	1.05 (0.84–1.30)	0.81 (0.65–1.01)
under weight vs. over weight	0.74 (0.49–1.12)	0.35 (0.17–0.72)	1.03 (0.60–1.76)
Smoking			
current *vs. *never smoker	0.90 (0.71–1.14)	0.79 (0.58–1.08)	1.05 (0.72–1.55)
former *vs. *never smoker	0.98 (0.72–1.32)	1.15 (0.78–1.70)	0.69 (0.42–1.13)
Alcohol drinking (No *vs. *yes)	0.97 (0.80–1.18)	0.95 (0.74–1.23)	0.89 (0.65–1.22)
Physical activity			
high *vs. *low	1.09 (0.81–1.46)	0.93 (0.62–1.39)	1.26 (0.81–1.95)
moderate *vs*. low	0.94 (0.70–1.28)	0.81 (0.53–1.23)	1.07 (0.68–1.68)
Educational level, years in school			
group 1 *vs. *3	1.24 (0.97–1.59)	1.38 (0.98–1.94)	0.91 (0.61–1.35)
group 2 *vs. *3	1.06 (0.86–1.30)	1.13 (0.84–1.53)	0.93 (0.69–1.26)
Marital status (no spouse *vs. *spouse)	1.25 (1.00–1.56)*	1.14 (0.84–1.55)	1.52 (1.07–2.15)*
Medical insurance (no *vs. *yes)	0.73 (0.59–0.90)*	0.84 (0.60–1.16)	0.77 (0.55–1.07)
Employment			
employed *vs. *retired	1.05 (0.86–1.28)	1.07 (0.80–1.43)	0.96 (0.72–1.28)
unemployed *vs*. retired	1.00 (0.81–1.25)	0.80 (0.59–1.08)	1.30 (0.93–1.81)
Social activity (no *vs. *yes)	0.80 (0.69–0.94)*	0.83 (0.66–1.03)	0.80 (0.64–1.00)*
Self rated health status (good *vs. *not good)	2.48 (2.08–2.96)*	2.25 (1.76–2.88)*	2.63 (2.03–3.41)*
Diabetes (no *vs. *yes)	1.01 (0.82–1.26)	1.10 (0.84–1.44)	0.98 (0.68–1.41)
Cardiovascular disease (no *vs. *yes)	1.47 (1.15–1.88)*	1.25 (0.92–1.71)	2.14 (1.41–3.26)*
Arthritis (no *vs. *yes)	1.23 (0.99–1.53)	1.40 (1.04–1.89)*	1.11 (0.81–1.53)
Depressive Symptoms (no *vs. *yes)	4.05 (3.12–5.26)*	3.91 (2.88–5.29)*	5.00 (2.94–8.52)*
Total hours of sleep/day	*	*	*
<7 *vs. *9< hrs	0.10 (0.07–0.14)*	0.10 (0.07–0.15)*	0.10 (0.05–0.17)*
7–9 *vs. *9< hrs	0.58 (0.42–0.78)*	0.64 (0.44–0.92)*	0.53 (0.31–0.92)*

Abbreviations: CES-D, Center for Epidemiologic Studies Depression Scale; OR, Odds ratio; CI, Confidence interval. *P < 0.05.

Group 1, 2 and 3 for educational level are 0–6, 7–9 and ≥10 years in school respectively.

The results of the multivariate analysis indicated that gender (Odds Ratio (OR) 0.51; 95% Confidence Interval (CI) 0.41 to 0.64), region (OR 0.61; 95% CI 0.51 to 0.73), medical insurance coverage (OR 0.73; 95% CI 0.59 to 0.90), social activity (OR 0.80; 95% CI 0.69 to 0.94), residential status (OR 1.27; 95% CI 1.01 to 1.60), SRH (OR 2.48; 95% CI 2.08 to 2.96), depressive symptoms (OR 4.05; 95% CI 3.12 to 5.26), CVD (OR 4.05; 95% CI 3.12 to 5.26) and sleep duration (<7 hours daily sleep (OR 0.10; 95% CI 0.07 to 0.14), 7–9 hours daily sleep (OR 0.58; 95% CI 0.42 to 0.78)) affected the quality of sleep independently from the effect of other variables in the model (Table 3).

When we stratified the analyses by region (Beijing/Shanghai) we found that gender, depressive symptoms, self-rated health and sleep duration still remained significantly associated with sleep quality in both north and south China. Residential status, marital status, social activity and present CVD only affected the sleep quality of participants living in Shanghai, while arthritis only affected those living in Beijing (Table 3).

After stratifying the analyses by gender and region simultaneously (Table 4), the effect of SRH, presence of depressive symptoms and sleep duration remained significant among all subgroups. Marital status, social activity, prostatism and residential status significantly affected Shanghai men and CVD significantly affected Beijing men and Shanghai women.

**Table 4:** Adjusted associated factors of sleep quality stratified by region and gender

	Association with sleep quality (OR with 95% CI)			
**Variable**	**Beijing-Male**	**Shanghai-Male**	**Beijing-Female**	**Shanghai-Female**

Residential location (rural *vs. *urban)	1.50 (0.92–2.45)	3.90 (2.06–7.39)*	0.68 (0.41–1.14)	0.86 (0.51–1.45)
Body Mass Index (BMI)				
normal vs. over weight	1.03 (0.73–1.47)	0.77 (0.53–1.11)	0.98 (0.73–1.32)	0.79 (0.60–1.05)
under weight vs. over weight	0.21 (0.08–0.57)*	1.27 (0.47–3.48)	0.42 (0.14–1.28)	0.91 (0.46–1.79)
Smoking				
current *vs. *never smoker	0.81 (0.52–1.27)	0.96 (0.62–1.48)	0.75 (0.47–1.22)	0.80 (0.24–2.66)
former *vs. *never smoker	1.16 (0.69–1.95)	0.63 (0.36–1.08)	1.06 (0.52–2.18)	0.17 (0.01–2.75)
Alcohol drinking (no *vs. *yes)	0.84 (0.59–1.19)	0.86 (0.58–1.26)	1.20 (0.81–1.77)	0.88 (0.44–1.75)
Physical activity				
high *vs. *low	0.99 (0.50–1.98)	2.09 (1.02–4.30)*	0.77 (0.46–1.30)	0.98 (0.55–1.75)
moderate *vs*. low	0.70 (0.34–1.42)	1.57 (0.75–3.31)	0.77 (0.45–1.32)	0.83 (0.45–1.50)
Educational level, years in school ‡				
group 1 *vs. *3	1.70 (0.98–2.96)	0.74 (0.37–1.50)	1.26 (0.79–1.99)	0.91 (0.54–1.52)
group 2 *vs. *3	1.05 (0.68–1.62)	0.82 (0.50–1.35)	1.18 (0.78–1.81)	0.82 (0.55–1.22)
Marital status (no spouse *vs. *spouse)	1.12 (0.64–1.96)	4.87 (2.12–11.19)*	1.30 (0.89–1.89)	1.08 (0.72–1.62)
Medical insurance (no *vs. *yes)	0.67 (0.42–1.09)	0.75 (0.44–1.28)	1.06 (0.66–1.71)	0.75 (0.48–1.16)
Employment				
employed *vs. *retired	0.82 (0.55–1.23)	1.10 (0.71–1.70)	1.34 (0.86–2.11)	0.93 (0.62–1.41)
unemployed *vs*. retired	0.77 (0.46–1.27)	1.29 (0.75–2.19)	0.96 (0.64–1.44)	1.68 (1.05–2.68)*
Social activity (no *vs. *yes)	0.86 (0.60–1.22)	0.64 (0.44–0.93)*	0.82 (0.61–1.10)	0.99 (0.74–1.33)
Self rated health status (good *vs. *not good)	2.18 (1.51–3.16)*	3.11 (2.00–4.83)*	2.36 (1.68–3.31)*	2.43 (1.75–3.39)*
Diabetes (no *vs. *yes)	1.05 (0.68–1.61)	0.78 (0.45–1.34)	1.15 (0.80–1.65)	1.11 (0.66–1.86)
CVD (no *vs. *yes)	1.78 (1.12–2.83)*	1.58 (0.85–2.96)	0.82 (0.53–1.25)	2.72 (1.47–5.04)*
Arthritis (no *vs. *yes)	1.74 (0.96–3.15)	0.56 (0.29–1.09)	1.35 (0.94–1.94)	1.31 (0.89–1.93)
Prostatism (no *vs. *yes)	1.17 (0.69–1.99)	1.72 (1.00–2.97)*		
Depressive Symptoms (no *vs. *yes)	3.13 (1.82–5.38)*	9.06 (3.50–23.49)*	4.34 (2.95–6.36)*	3.44 (1.77–6.66)*
Total hours of sleep/day	*	*	*	*
<7 *vs. *9< hrs	0.12 (0.06–0.23)*	0.11 (0.04–0.29)*	0.08 (0.04–0.14)*	0.08 (0.04–0.16)*
7–9 *vs. *9< hrs	0.66 (0.37–1.16)	0.61 (0.23–1.62)	0.64 (0.39–1.06)	0.45 (0.22–0.90)*

Abbreviations: CES-D, Center for Epidemiologic Studies Depression Scale; CVD, Cardio-Vascular Disease; OR, Odds ratio; CI, Confidence interval. *P < 0.05.

‡ Group 1, 2 and 3 for educational level are 0–6, 7–9 and ≥10 years in school respectively.

### Sleep quality and cardio-metabolic biomarkers

Significant differences in the level of resistin, PAI-1, insulin, HOMA-IR, HOMA2-IR, LDL, TCH and TG were observed between different levels of sleep quality (Table 5). When we analyzed these associations among a subsample of the population who were free from CVD and diabetes, we found that significant associations remained for insulin, HOMA-IR, HOMA2-IR, LDL, TCH and TG (data not shown).

**Table 5:** Cardio-metabolic factors and biomarkers by levels of sleep quality

**Variable, mean (SD)**	**Total**	**well**	**common**	**poor**	**p-value**
Resistin	11.47(9.37)	11.26 (8.92)	11.24 (8.92)	12.57 (11.34)	0.0428*
PAI-1	14.74(18.17)	14.35 (18.71)	15.44 (17.56)	14.71 (17.50)	0.0011*
RBP4	40.11(11.74)	39.78 (11.59)	40.48 (11.58)	40.47 (12.48)	0.2924
Adiponectin	16.48(11.62)	16.47 (11.58)	16.06 (11.41)	17.29 (12.11)	0.1914
IL-6	1.55(2.78)	1.46 (2.13)	1.68 (3.63)	1.61 (2.84)	0.4680
CRP	1.58(4.30)	1.42 (2.29)	1.72 (5.54)	1.82 (6.24)	0.9505
Insulin	15.46(9.50)	15.00 (9.37)	16.07 (8.86)	15.81 (10.93)	<0.0001*
HOMA-IR	4.10(3.36)	3.95 (3.36)	4.32 (3.25)	4.14 (3.55)	<0.0001*
HOMA2-IR	0.31(0.22)	0.29 (0.19)	0.32 (0.27)	0.31 (0.22)	<0.0001*
SBP	140.11(22.49)	139.5 (22.06)	140.7 (22.94)	141.1 (22.99)	0.2338
DBP	80.16(10.79)	80.18 (10.74)	80.15 (10.82)	80.11 (10.92)	0.9892
HDL	1.28(0.33)	1.27 (0.33)	1.27 (0.33)	1.30 (0.36)	0.5415
LDL	3.26(0.97)	3.19 (0.94)	3.36 (0.99)	3.30 (1.01)	<0.0001*
TCH	4.70(0.98)	4.63 (0.97)	4.79 (0.97)	4.75 (1.02)	0.0001*
TG	1.39(1.07)	1.36 (1.07)	1.44 (1.12)	1.39 (0.96)	0.0490*

Abbreviations: PAI-1, plasminogen activator inhibitor-1; RBP4, retinol binding protein 4; IL-6, interleukin-6; IL6, interleukin-6; CRP, C-reactive protein; HOMA-IR, homeostasis model assessment of insulin resistance (fasting glucose (mmol/L) × fasting insulin (μU/ml)/22.5); SBP, systolic blood pressure; DBP, diastolic blood pressure; HDL, high-density lipoprotein; LDL, low-density lipoprotein; TCH, total cholesterol; TG, triglycerides; SD, standard deviation;

## Discussion

Only half of middle aged and elderly Chinese in our study reported good sleep quality. Quality of sleep varied substantially by gender and geographical location and was affected by level of self-rated health, presence of depressive symptoms, sleep quantity, chronic diseases and social activity. Significant associations between levels of sleep quality and resistin, PAI-1, insulin, HOMA-IR, HOMA2-IR, LDL, TCH and TG were observed. These associations remained significant for insulin, HOMA-IR, HOMA2-IR, LDL and TCH among a subgroup of participants who were free of CVD and diabetes.

Sixteen percent of the elderly Chinese assessed their sleep quality as poor in this study. They showed higher prevalence of mental and physical diseases (depression, CVD, arthritis, prostatism (men only)) and shorter daily sleep time compared to participants that reported a good level of sleep quality. We also observed significant gender differences in those who reported poor sleep quality wherein Chinese women were almost 50% less likely to report good sleep quality than men. Similar to our findings, Li et al.[6] found a 12% prevalence of insomnia among Hong Kong Chinese adults. Li et al.[6] studied 9851 Hong Kong Chinese aged 18–65 years and found a 60% greater chance of insomnia being reported among women than men. Klink et al.[5] also found there was a 50% higher chance of women reporting insomnia. Furthermore, in a study of 400 adults aged 20–70 years, Reyner et al.[7] found women, reported more awakenings, more total time spent awake during the night and poorer sleep quality. This difference in sleep quality could be due to gender differences in the prevalence of psychiatric morbidities, socio-cultural factors and coping strategies. [5-7]

We also found that sleep quality related to geographical location. Those living in the north were 40% less likely to report good sleep quality compared to those living in the south. This could be due to differences between the two cities wherein compared to Shanghai residents, Beijing residents show almost twice the prevalence of chronic diseases (depression, CVD, diabetes and metabolic syndrome), higher levels of alcohol consumption (37% Beijing vs. 20% Shanghai) and smoking (31% Beijing vs. 25% Shanghai), higher prevalence of nap taking (55% Beijing vs. 29% Shanghai) and less medical insurance coverage (53% Beijing vs. 86% Shanghai). To our knowledge this is the first study investigating differences in sleep quality as a function of geographical and residential status in China.

The effect of SRH (which is considered to be a valid representative of physical and mental health[38-40]) was substantial on sleep quality for the total population and for gender and geographical subgroups. Those who reported good/excellent SRH had twice the chance of reporting good sleep quality compared to those that had fair, poor or very poor SRH.

In our study, we also found a strong association between sleep quality and presence of depressive symptoms, with the strongest effect among Shanghai men and Beijing women. The likelihood of reporting poor sleep quality was three times higher in the presence of depressive symptoms. Our findings are consistent with other studies that have shown significant associations between depression and insomnia[41] and depression and sleep disturbances.[42] The association between poor sleep and depression has also been observed in an elderly population.[2]

Currently, the number of hours required for optimal functioning in an elderly population is not known. Although it is commonly believed that 8 hours of sleep per night is optimal for good health, recent studies suggest that mortality risk is lower among those sleeping 7 hours.[21] The total hours of daily sleep includes night time sleep and nap time (nap taking is common mainly in north of China) and for that reason, 7–9 hours was categorized as normal daily sleep hours in our study. We found a substantial effect of sleep quantity on quality of sleep. Having a short sleep time significantly decreased the chance of reporting good quality of sleep. Even those reporting 7–9 hours of daily sleep were less likely to report good sleep quality than those reporting more than 9 hours of daily sleep. Presence of CVD also affected the levels of sleep quality among the total population but when we stratified by gender and residential location this effect remained significant only among Beijing men and Shanghai women.

We did not find any significant association between sleep quality and different lifestyle characteristics (smoking and alcohol drinking habits), age and BMI. This is consistent with Elwood et al. [38] who studied 1986 welsh men aged 55–69 y and found no significant association between prevalence of insomnia and age, BMI, smoking and alcohol drinking habits. However, it is possible that the effect of smoking and alcohol drinking in our analysis might be confounded with gender as the majority of Chinese smokers and drinkers were men. Furthermore, this is affected by the limited number of participants in the extreme categories of alcohol consumption and BMI, which limits the statistical power to identify a potential association between these two variables and sleep patterns in our population.

Despite lower socio-economic status (lower average income, fewer years in full-time education and less medical insurance coverage) rural residents were more likely to report good levels of sleep quality compare to urban residents. This could be due to differences in lifestyle wherein those who live in rural areas tend to have higher levels of physical activity, higher age of retirement, less stressful lifestyles and a lower prevalence of both CVD (7.7% rural vs. 13% urban) and diabetes (11.4% rural vs. 17% urban).

In our analyses of the total sample and subgroups of the sample, we found no significant effects of income and education. This could be due to the fact that residence is a more comprehensive factor that might partially confound the effect of socio-economic factors (i.e. our sample of the Chinese population is skewed towards lower income and education and late retirement in rural areas).

Concentrations of inflammatory biomarkers, plasma insulin and index of insulin resistance were associated with sleep quality for the total population and among a subgroup of participants that were free from CVD and diabetes. However, no clear trends in the levels of biomarkers as a function of the different levels of sleep quality (good/common/poor) were observed. This is the first study to evaluate the associations between sleep quality and inflammatory markers and measures of insulin resistance in China and to evaluate the distribution and factors associated with sleep quality in such a vast country which has many geographical and residential differences

Although we considered the effect of comprehensive measurements of lifestyle, socio-demographic factors and presence of disease, our findings were limited. This could be due to the cross-sectional nature of our data and further longitudinal studies should be carried out to confirm our findings.

Furthermore, sleep patterns were measured using a questionnaire; a criticism of this type of measurement is that answers are provided by the participants and can be affected by recall bias. Furthermore, due to the size of the population and the extent of the general questionnaire, for logistic reasons the questions that we used are not as thorough as alternative validated questionnaires designed to evaluate sleep disturbances (e.g The Pittsburg Sleep Quality Inventory). Nevertheless, these factors could only generate a non-differential misclassification of the exposure (sleep patterns) that would attenuate the size of the effects hereby reported and the level of sleep disturbances in the population studied, but not necessarily our results and conclusions. Prospective evaluations and studies that provide more sensitive measures of sleep patterns in Chinese populations are required to clarify the determinants that affect sleep quality. This will also allow us to assess whether sleep disturbances are associated with early deviations from adequate cardio-metabolic health.

## Conclusion

In general, only half of the population reported good sleep quality. Compared to their counterparts, men and people living in south China were twice as likely to report good sleep quality. Both physical and mental health significantly affected the quality of sleep in elderly Chinese and having enough daily sleep hours substantially increased the likelihood of reporting better sleep quality. Although we did not find any clear trends between levels of sleep quality and levels of inflammatory markers, we did find a significant association between levels of sleep quality and resistin, insulin, HOMA-IR, HOMA2-IR, LDL, and TCH. These associations were found among the total population and a subgroup of participants free from CVD and diabetes.

Considering the alarming increase of sleep disturbances that modern societies are experiencing and the deleterious consequences that a poor night sleep can exert on our metabolism and health, it is fundamental to clarify the factors that might generate sleep disturbances, how these disturbances can affect the biological homeostasis and identify potential interventions to prevent sleep disturbances at an early stage.

## Abbreviations

ADIPO: adiponectin; ANOVA: analysis of variance; BMI: body mass index; CI: confidence interval; CRP: C-reactive protein; CVD: cardiovascular disease; DBP: diastolic blood pressure; HDL: high-density lipoprotein; IL-6: interleukin-6; LDL: low-density lipoprotein; MetS: metabolic syndrome; OR: odds ratio; PAI-1: plasminogen activator inhibitor-1; RBP4: retinol-binding protein 4; SBP: systolic blood pressure; SD: standard deviation; SRH: self rated health; TG: triglycerides; TCH: total cholesterol.

## Competing interests

The authors declare that they have no competing interests.

## Authors' contributions

All authors mentioned participated actively in all and each of the following aspects for this article: Conception and design, or analysis and interpretation of data, drafting the article or revising it critically for important intellectual content and final approval of the version to be published.

## Pre-publication history

The pre-publication history for this paper can be accessed here:


